# High-fat diet-induced intestinal dysbiosis is associated with the exacerbation of Sjogren’s syndrome

**DOI:** 10.3389/fmicb.2022.916089

**Published:** 2022-07-22

**Authors:** Minjie Zhang, Yichen Liang, Yanbo Liu, Yixuan Li, Long Shen, Guixiu Shi

**Affiliations:** ^1^Department of Rheumatology and Clinical Immunology, The First Affiliated Hospital of Xiamen University, Xiamen, China; ^2^Xiamen Key Laboratory of Rheumatology and Clinical Immunology, Xiamen, China; ^3^Oncology Department, Northern Jiangsu People's Hospital, Yangzhou, China; ^4^Cancer Institute Affiliated to Northern Jiangsu People's Hospital, Yangzhou, China; ^5^Medical College, Yangzhou University, Yangzhou, China; ^6^Department of Ophthalmology, Xiang’an Hospital of Xiamen University, Xiamen, China; ^7^Fujian Provincial Key Laboratory of Ophthalmology and Visual Science, Xiamen University, Xiamen, China; ^8^Eye Institute of Xiamen University, Xiamen, China; ^9^School of Medicine, Xiamen University, Xiamen, China

**Keywords:** primary Sjögren’s syndrome, high-fat diet, gut microbiota, lacrimal gland, inflammation

## Abstract

Environmental factors are believed to influence the evolution of primary Sjögren’s syndrome (pSS). The aims of this study were to investigate the association of pSS with a high-fat diet (HFD) and to relate HFD-induced gut dysbiosis to pSS exacerbation. Male Wild Type (WT) and IL-14α transgenic mice (IL-14α TG) were fed a standard diet (SD) and HFD for 11 months. We found an increase in the autoantibody level, more severe dry eye, severe dry mouth symptoms, and an earlier presence of systemic features in the IL-14α TG mice treated with HFD. These data suggest that HFD can promote the process of pSS in the IL-14α TG mice. In addition, an HFD leads to a decrease in the richness of gut microbiota of IL-14α TG mice treated with HFD. The abundance of *Deferribacterota* was significantly enriched in the IL-14α TG mice treated with HFD compared with other groups. Through the mental test between gut microbiota and clinical parameters, we found that HFD-induced dysbiosis gut microbiota were associated with pSS clinical parameters. In conclusion, HFD results in the aggravation of pSS progression, likely due to the increase of potentially pathogenic microorganisms.

## Introduction

Primary Sjögren’s syndrome (pSS) is a chronic autoimmune disorder characterized by lymphocytic infiltration of lacrimal and salivary glands, leading to dry mouth and eyes. Moreover, a third of patients develop inflammation in numerous extraglandular sites, including the renal tract, the lung tract, and the gastrointestinal tract (Seror et al., 2010; Ramos-Casals et al., 2014). However, the pathogenesis of pSS remains to be clarified. The evolution of pSS is believed to be influenced by environmental and genetic factors (Jonsson, 2022). For decades, with the increased intake of high-fat food, high-fat diet (HFD)-related diseases have attracted great attention. It is well known that HFD contributes to systemic low-grade inflammation in the body (Duan et al., 2018). Furthermore, many studies reported that HFD-induced inflammation can exacerbate the progression of autoimmune diseases, such as multiple sclerosis (Schreiner and Genes, 2021), systemic lupus erythematosus (SLE; Hanna Kazazian et al., 2019), and rheumatoid arthritis (RA; Poudel et al., 2020). However, there are few studies elucidating the relationship between HFD and pSS.

Several studies showed that HFD can change the composition of gut microbes. Moreover, these studies reported that intestinal dysbiosis induced by HFD plays an important role in diseases, such as hepatic disease (Mouries et al., 2019), colorectal tumorigenesis (Yang et al., 2022), and retinal degeneration (Kutsyr et al., 2021). *Firmicutes/Bacteroidetes* ratio is usually used as a marker of microbiome dynamics to reflect the change of gut bacteria species (Mariat et al., 2009). HFD leads to an increase in *Firmicutes/Bacteroidetes* ratio (Jiao et al., 2018). Bile acid (BA) secretion is increased in the intestinal tract, which is related to HFD-associated gut dysbiosis. The increased secretion of BAs leads to intestinal permeability and inflammation (Gerard, 2013). Moreover, gut dysbiosis caused by HFD also results in increased LPS production and translocation (Cani et al., 2007; Kim et al., 2012). The increase of HFD-induced circulating LPS level tends to stimulate TLRs around the body, resulting in cytokine release and inflammatory response (Kim et al., 2012). Fecal transplantation from HFD mice to germ-free mice leads to NF-κB pathway activation, confirming that HFD-induced intestinal dysbiosis is enough to cause inflammation (Ding et al., 2010).

The association between intestinal microbiota and pSS has recently become a research focus. Mandl et al. and de Paiva et al. reported that intestinal dysbiosis was associated with worse ocular and systemic disease severity index (de Paiva et al., 2016; Mandl et al., 2017). The alteration of gut microbiome composition contributed to pSS patient’s inflammatory processes through increased production of proinflammatory cytokines and decreased release of the anti-inflammatory cytokine (Cano-Ortiz et al., 2020). Butyrate, the metabolite of *Faecalibacterium*, which is reduced in the pSS patients’ gut microbiome, can ameliorate SS (Kim et al., 2021). Environmental factors such as diet may modify the gut microbiome composition.

Thus, we hypothesize that HFD may promote the evolution of pSS through gut dysbiosis. To confirm the hypothesis, we used IL-14α transgenic mice (IL-14α TG), a mouse model that mimics the clinical features of pSS in the same time frame as in humans. This mouse model not only shows lacrimal gland and salivary gland inflammation but also shows systemic manifestations of pSS (Shen et al., 2009). The IL-14α TG mice were fed with HFD at 11 months and then were examined for intestinal microbial composition and clinical, serological, and histological features of pSS.

## Materials and methods

### Animal

IL14α TG mice were gifts from Dr. Julian L. Ambrus, Jr.’s Laboratory at the State University of New York at Buffalo. The detailed characterizing data of the IL14α TG mice could be found in the previous publications (Shen et al., 2006, 2009). IL14α TG mice and WT mice were housed and bred at the Experimental Animal Center of Xiamen University. Mice were fed two different diets. Standard diet (SD, 10 kCal% fat, 1,022; Beijing HFK Bioscience, Beijing, China) groups were fed SD all the time. HFD (60 kCal% fat, D12492; Research Diets, New Brunswick, NJ, United States) groups were treated with HFD from 4 weeks of age. All of them were sacrificed at 12 months of age.

### Evaluation of salivary gland and lacrimal gland secretions

Salivary gland secretions were determined by suctioning of saliva for 15 min after pilocarpine injection, as previously described (Shen et al., 2010). Tear production was measured using the phenol red impregnated cotton threads (Zone-Quick, Yokota, Tokyo, Japan) at the same time point (9:00 a.m.). Before each measurement, an absorbent paper was used to remove the tear fluid inside the conjunctival sac to avoid any contribution from the lacrimal lake. A thread was then placed in the lower conjunctival fornix for 15 s at a position that was one-third of the distance between the lower eyelid and the lateral canthus. The distance that the red color dye front migrated along the thread length was recorded in millimeters.

### Determination of serum autoantibodies

Antinuclear antibodies (ANA) in the mouse serum were tested by HEp-2 and monkey liver tissue substrate (EUROIMMUN). The serum was diluted with PBS containing 0.1% Tween 20 at 1:20, 1:40, 1:80, 1:160, and 1:320. Thirty microliter serum dilutions were dropped onto the reaction carrier coated with HEP2 cells for 30 min reaction at room temperature. Slides were washed with PBS containing 0.1% Tween 20 two times. The sections were then incubated with a goat Anti-Mouse IGG Secondary Antibody FITC (1:100, 2 mg/ml; Invitrogen, Waltham, MA, United States) for 30 min at room temperature. After washing two times with PBS, the embedding medium was placed onto a cover glass. Digital images were captured with a Leica upright microscope (DM2500; Leica Microsystems, Wetzlar, Germany) at the same exposure conditions. ANA titer is the highest dilution that reveals a positive fluorescent signal.

### Slit-lamp evaluation and fluorescein test

The corneas were imaged under a slit-lamp microscope (Takagi Seiko Co., Ltd., Nagano, Japan) by a single masked ophthalmologist. After that, 1 μl of 1% liquid sodium fluorescein (Jingmingxin Co., Ltd., Tianjin, China) was dropped into the conjunctival sac, and corneal epithelial fluorescein staining was recorded 90 s later under the slit-lamp microscope with a cobalt blue filter. The grade of corneal damage was designated based on previously reported criteria (Lin et al., 2011). For grading of the fluorescein staining, the cornea was divided into four quadrants, staining was scored separately, and the scores of four quadrants were summed and analyzed. The four scores were added to arrive at a final grade (total, 16 points). The fluorescein score was analyzed as follows: absent, 0; slightly punctate staining less than 30 spots, 1; punctate staining more than 30 spots, but not diffuse, 2; severe diffuse staining but no positive plaque, 3; and positive fluorescein plaque, 4.

### Histological examination

Submandibular glands (SMG), lacrimal glands (LG), the lungs, and the kidneys were removed. Tissue sections were prepared and stained by standard histological techniques using H&E as previously described (Shen et al., 2010). All histopathologic scoring was performed using a blind method.

Focus Score (FS) was used to evaluate the lymphocyte infiltration grade in the SMG and LG. FS refers to the number of foci per 4 mm^2^ over the whole glandular area. It is calculated by the number of foci divided by the whole glandular surface area in mm^2^ and multiplied by 4 (Fisher et al., 2017). For lung, interstitial pneumonitis was evaluated microscopically through the infiltration area of the lymphocyte. Histologic score (HS) depends on the percentage of the infiltration area on the sections of lungs as follows: normal, score 0; 10–30%, score 1; 30–60%, score 2; and >60%, score 3 (Wakasa-Morimoto et al., 2008). For the kidney, the damage of the kidney includes tubular necrosis, loss of brush border, cast formation, tubular dilatation, and immune cell infiltration. Histologic score (HS) depends on the percentage of the damaged area on the sections of kidney as follows: normal, score 0; 0–10%, score 1; 11–25%, score 2; 26–45%, score 3; 46–75%, score 4; and >76%, score 5 (Lu et al., 2019).

### DNA extraction and PCR amplification

Total microbial genomic DNA was extracted from mouse feces using the E.Z.N.A.® soil DNA Kit (Omega Bio-Tek, Norcross, GA, United States) according to the manufacturer’s instructions. The quality and concentration of DNA were determined by 1.0% agarose gel electrophoresis and a NanoDrop® ND-2000 spectrophotometer (Thermo Scientific Inc., United States). Then, the DNA was stored at −80°C before further use. Sequencing of the PCR-amplified V3–V4 of the bacterial 16S rRNA gene was performed using an ABI GeneAmp® 9700 PCR thermocycler (ABI, CA, United States) at Majorbio Bio-Pharm Technology Co., Ltd. (Shanghai, China). The primers used are as follows: 338F (5′-ACTCCTACGGGAGGCAGCAG-3′) and 806R (5′-GGACTACHVGGGTWTCTAAT-3′; Liu et al., 2016). PCR was performed in a reaction mixture containing 4 μl 5 × Fast Pfu buffer, 2 μl 2.5 mM dNTPs, 0.8 μl each primer (5 μM), 0.4 μl Fast Pfu polymerase, 0.2 μl BSA, 10 ng of template DNA, and ddH_2_O to a final volume of 20 μl. The reaction condition was as follows: initial denaturation at 95°C for 3 min, followed by 27 cycles of denaturing at 95°C for 30 s, annealing at 55°C for 30 s, extension at 72°Cfor 45 s, and single extension at 72°C for 10 min and end at 4°C. All samples were amplified in triplicate.

### DNA purification and sequencing

The PCR product was extracted from 2% agarose gel and purified using the AxyPrep DNA Gel Extraction Kit (Axygen Biosciences, Union City, CA, United States) according to the manufacturer’s instructions and quantified using Quantus™ Fluorometer (Promega, United States). Then, the purified amplicons were pooled in equimolar amounts and paired-end sequenced on an Illumina MiSeq PE300 platform/NovaSeq PE250 platform (Illumina, San Diego, United States).

### Data processing

Raw FASTQ files were de-multiplexed using an In House Perl Script and then quality-filtered by Fastp (v0.19.6; Chen et al., 2018) and merged by FLASH (v1.2.11; Magoč and Salzberg, 2011). The filtering criteria were as follows: (i) the 300 bp reads were truncated at any site receiving an average quality score of <20 over a 50-bp sliding window, and the truncated reads shorter than 50 bp and reads containing ambiguous characters were discarded; (ii) only overlapping sequences longer than 10 bp were assembled according to their overlapped sequence. The maximum mismatch ratio of the overlap region is 0.2. Reads that could not be assembled were discarded. (iii) Samples were distinguished according to the barcode and primers, and the sequence direction was adjusted, with exact barcode matches and two nucleotide mismatches in primer matching. Then, the optimized sequences were clustered into operational taxonomic units (OTUs) using UPARSE (v7.0; Edgar, 2013) with a 97% identity threshold. Representative sequences of OTUs were obtained, and all sequences were blasted against the SILVA database (v138) using an RDP classifier (v 11.5). The confidence threshold was set to 0.7. Due to the variation in sequence depths among samples, all samples were normalized to the lowest depth by subsampling (30, 246 reads per sample). Based on Bergey’s taxonomy, the OTU sequences are classified into six levels.

### Comparison of gut communities and Bioinformatics analysis

Based on the OTUs information, alpha diversity indices, including the Shannon index and Chao1 richness, were calculated with the QIIME software package (Caporaso et al., 2010). The similarity among the microbial communities in different samples was determined by principal coordinate analysis (PCoA) based on Bray–Curtis dissimilarity using vegan packages and QIIME software package (Caporaso et al., 2010). The different groups were statistically compared by using the similarity analysis (ANOSIM). The overlap of the microbial communities was determined by the R values from the ANOSIM, according to the method from Buttigieg and Ramette (2014), with *R* > 0.75 indicating well separated, 0.50 < *R* ≤ 0.75 indicating separated but overlapped, 0.25 < *R* ≤ 0.50 indicating separated but strongly overlapped, and 0.25 = *R* indicating barely separated.

The linear discriminant analysis (LDA) effect size (LEfSe; Segata et al., 2011)^1^ was performed to identify the significantly abundant taxa (phylum to genera) of bacteria among the different groups (LDA score > 2, *p* < 0.05).

The co-occurrence networks were constructed to explore the internal community relationships across the samples (Barberán et al., 2012). To visualize the associations in the network, we constructed a correlation matrix by calculating the possible pairwise Spearman’s rank correlations. A correlation between two nodes was considered statistically robust if Spearman’s correlation coefficient was over 0.6 or less than −0.6 and the value of *p* was <0.01(Jiang et al., 2017a). The nodes in the reconstructed network represented bacterial taxa (OTUs), and the edges represented the high and significant correlations between the nodes. Network analyses were performed using the igraph, vegan, and Hmisc packages in R software (Jiang et al., 2017a). The correlation networks were visualized using the Gephi software.

The Mantel test was performed to detect the correlation between clinical parameters and gut microbial communities in different groups using the ggcor R package.

All analyses were conducted in R (version 3.5.1, R Development Core Team) unless otherwise stated.

### Statistical analysis

Data were analyzed using GraphPad Prism 7.0 software and R Stats Software. Significant differences between groups were compared by the one-way ANOVA, the Wilcoxon rank-sum test, the analysis of similarities (ANOSIM), or the Kruskal-Wallis test. Correlation analysis between gut microbiota and clinical parameters was performed using Spearman’s correlation. All results were expressed as the mean ± SD and a value of *p* < 0.05 was considered a significant difference.

## Results

### HFD exacerbated dry mouth manifestation and increased autoantibody levels in the IL-14α TG mice

**Figure 1:**
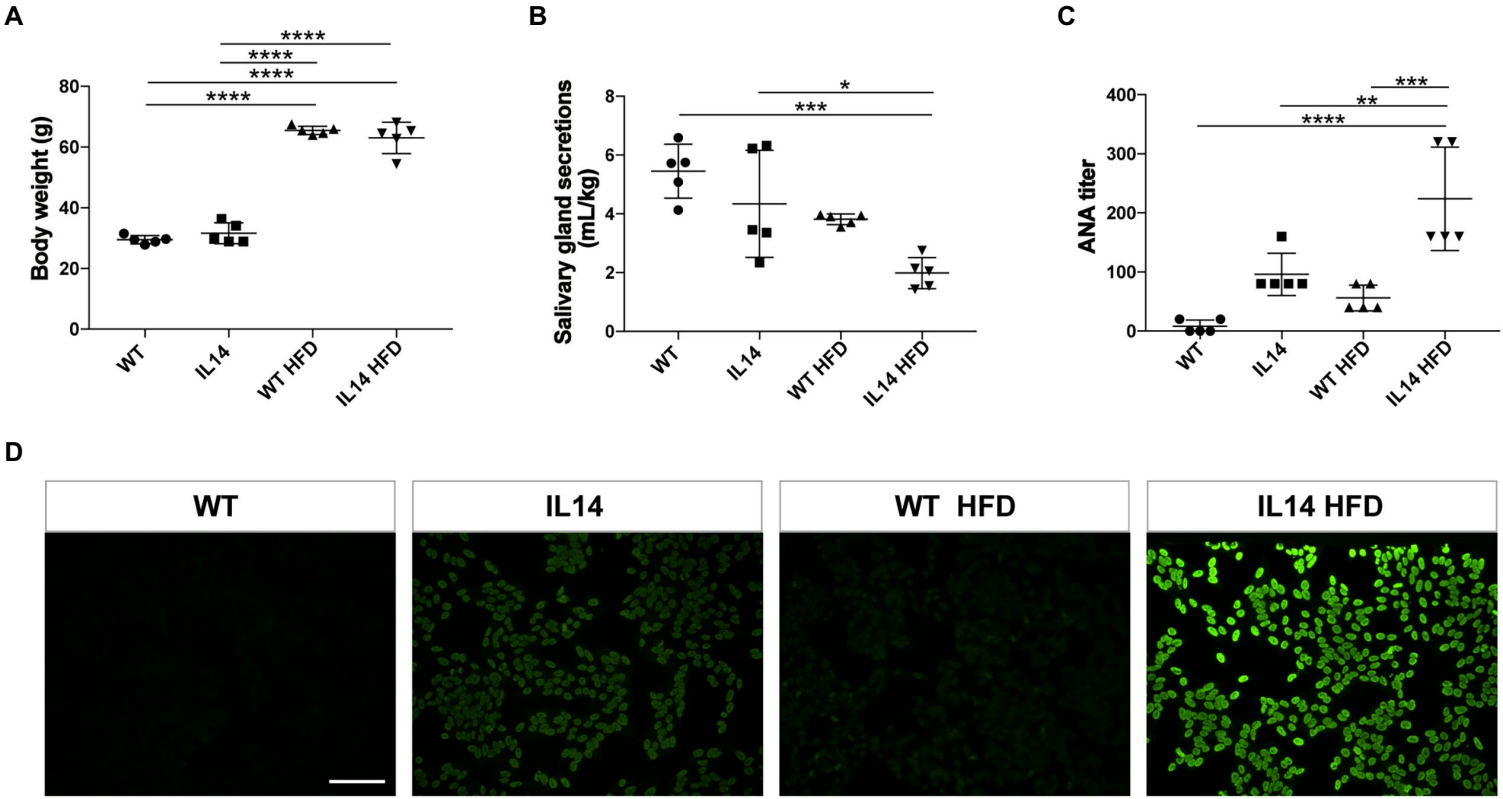
High-fat diet (HFD) increased autoantibody level and exacerbated dry mouth manifestation in the IL-14α TG mice. **(A)** The body weight was increased after HFD. **(B)** Stimulated salivary gland secretions were measured and normalized to the body weight. **(C)** The results of the antinuclear antibodies (ANA) titer showed that the IL14 HFD group was significantly higher than the other three groups. **(D)** Hep-2 cells were directly stained by serum at 1:80 dilutional titer. ^*^p ≤ 0.05; ^**^p ≤ 0.01; ^***^p ≤ 0.005; and ^****^p ≤ 0.001; *n* = 5 per group. Scale bars: 200 μm for fluorescent staining.

The body mass of WT HFD and IL14 HFD mice had become significantly higher than that of WT and IL-14α TG mice (Figure 1A). Dry mouth is a remarkable clinical symptom of pSS. We next investigated the functional changes in salivary gland secretions. The salivary gland secretions of the IL14 HFD mice were significantly different from the salivary gland secretions of the WT group and the IL14 group (Figure 1B). The presence of autoantibody is one of the characteristic features of pSS. Antinuclear antibodies (ANA) are a traditional biomarker for pSS diagnosis (Stefanski et al., 2017). The result of indirect immunofluorescence on HEp-2 cells showed that the titer of the IL14 HFD group was significantly higher than that of the IL14 group and the WT HFD group (Figures 1C,D). This suggests that HFD can affect autoantibody production and salivary gland secretions in the IL-14α TG mice.

### HFD promoted dry eye symptoms in the IL-14α TG mice

**Figure 2:**
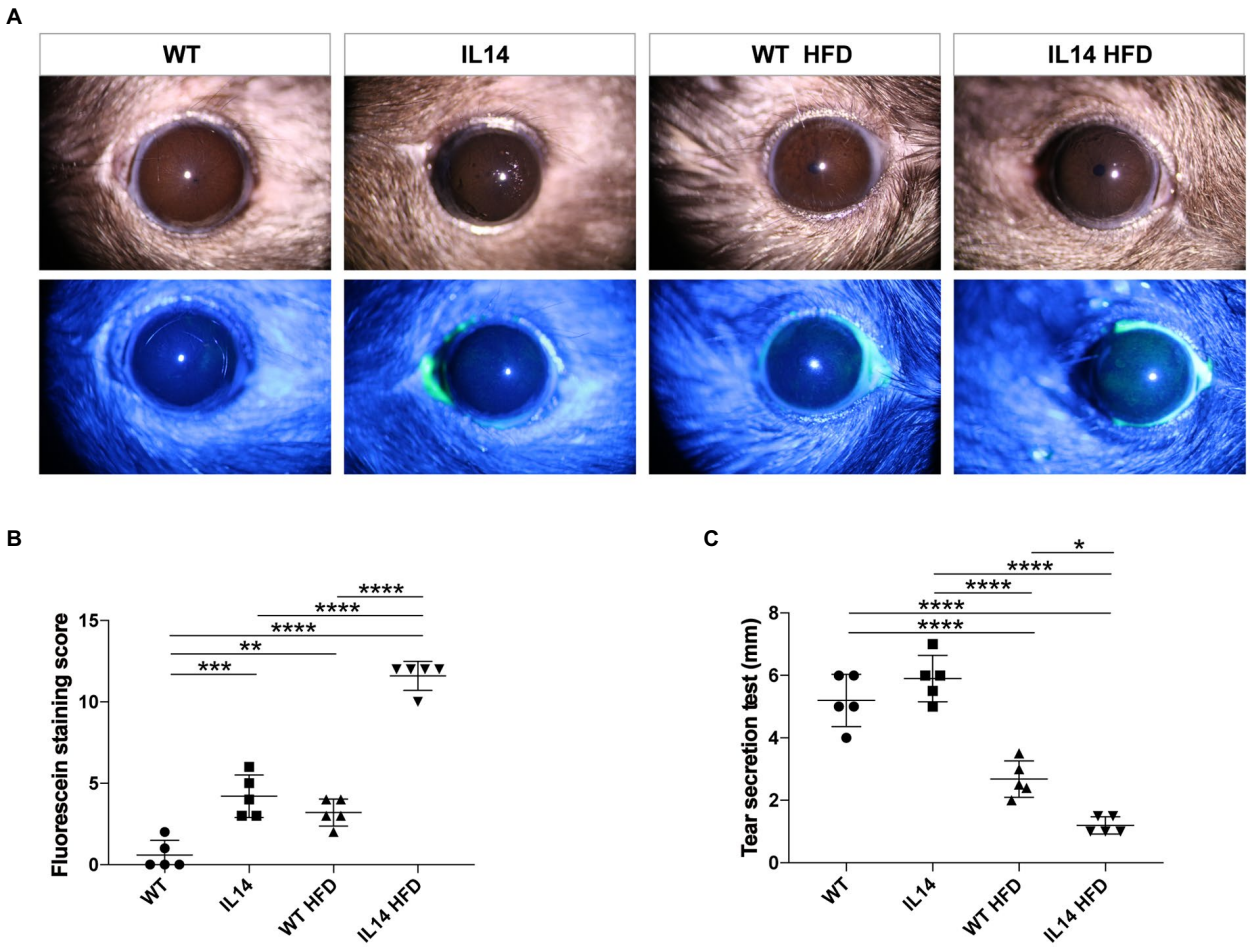
HFD aggravated dry eye-like ocular surface damage in the IL-14α TG mice. The ocular surfaces of the WT, IL14, WT HFD, and IL14 HFD groups were compared using **(A)** slit-lamp observation and fluorescence staining. **(B)** The fluorescein staining score showed that the IL14 HFD group was dramatically higher than the other three groups. **(C)** Tear secretion was measured by the phenol red thread test. ^*^p ≤ 0.05; ^**^p ≤ 0.01; ^***^p ≤ 0.005; and ^****^p ≤ 0.001; *n* = 5 per group.

Slit lamp and corneal fluorescein staining images showed no obvious changes in the corneas of the WT and the WT HFD groups. However, there was a notable amount of punctate corneal staining in the IL14 and WT HFD groups. Corneal fluorescein staining increased remarkably in the IL14 HFD group compared to other groups (Figures 2A,B). Tear production, moreover, was significantly decreased in both WT HFD and IL14 HFD groups compared with comparable SD groups (Figure 2C). These findings suggested that an HFD aggravated dry eye-like pathologic changes in the ocular surface of the IL-14α TG mice.

### Gland involvement worsened in the IL-14α TG mice after HFD

**Figure 3:**
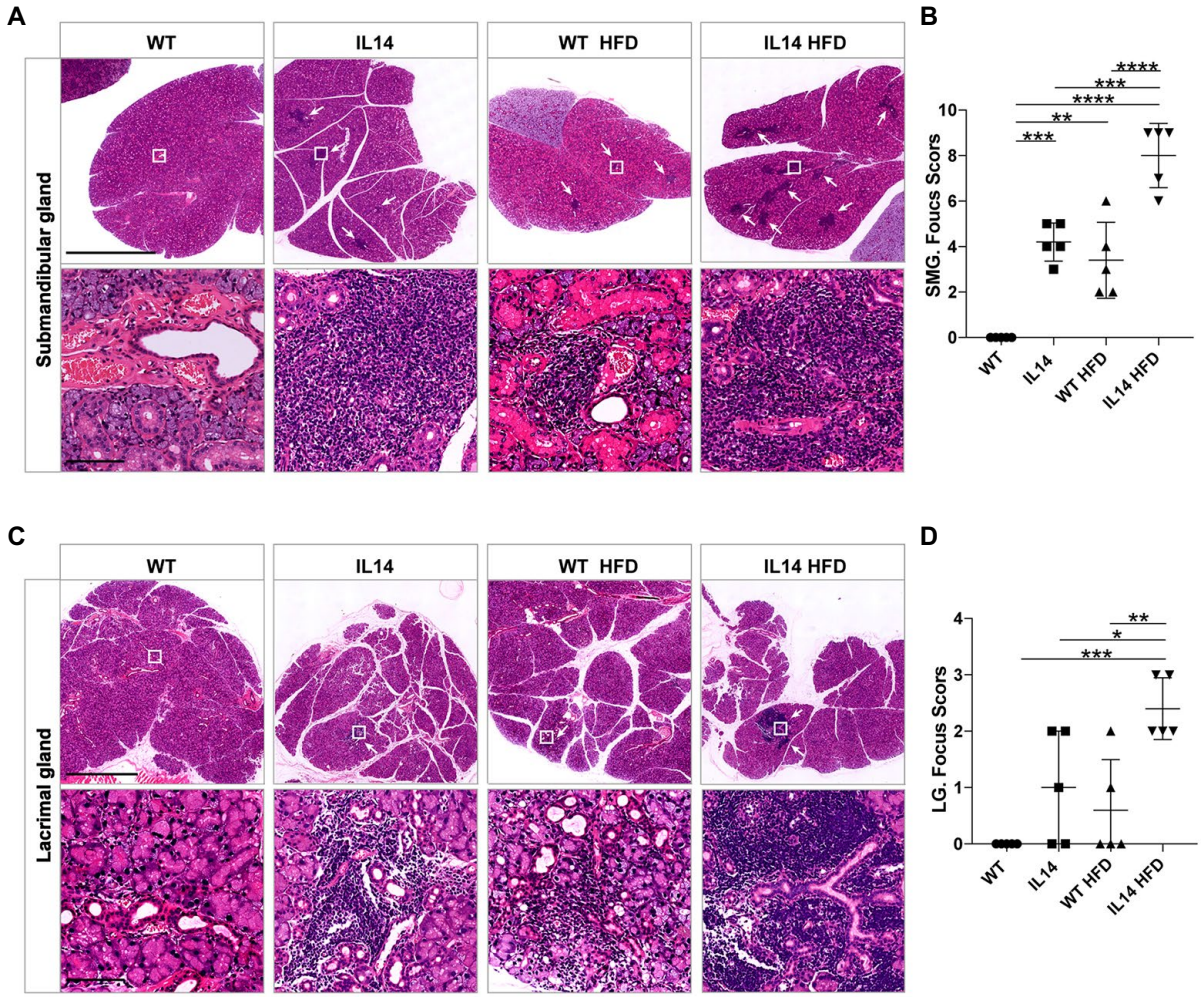
HFD exacerbated submandibular and lacrimal glands inflammation in the IL-14α TG mice. SMG and LG tissues from the WT, IL14, WT HFD, and IL14 HFD groups were isolated for analysis. SMG **(A)** and LG **(C)** sections were stained with H&E, and representative images are shown. **(B,D)** Focus score of SMG and LG was quantified as described in the section “Materials and Methods,” showing a significant increase in infiltration after HFD. ^*^p ≤ 0.05; ^**^p ≤ 0.01; ^***^p ≤ 0.005; and ^****^p ≤ 0.001; *n* = 5 per group. SMG, Submandibular gland; LG, Lacrimal gland. Scale bars: 2.5 mm for H&E staining of SMG, 100 μm for enlarged images of SMG, 1 mm for H&E staining of LG, and 100 μm for enlarged images of LG. White arrowheads indicate the site of lymphocyte infiltration.

The involvement of the lacrimal gland and salivary gland is one of the hallmarks of pSS. To study the development of organ injury, mice were autopsied in different groups. The injury to the submandibular glands (SMG) and lacrimal glands (LG) occurs before 12 months of age (Yang et al., 2022). We therefore investigated the histology of the LG and SMG in four groups at 12 months of age. The results showed that the IL14 group developed the same SMG and LG injuries as the WT HFD group. However, the IL14 HFD group showed more extensive and severe lymphocytic inflammatory infiltration of SMG and LG, as indicated by the higher focus scores (Figures 3A–D).

### Extra-glandular features appeared earlier in the IL-14α TG mice after HFD

**Figure 4:**
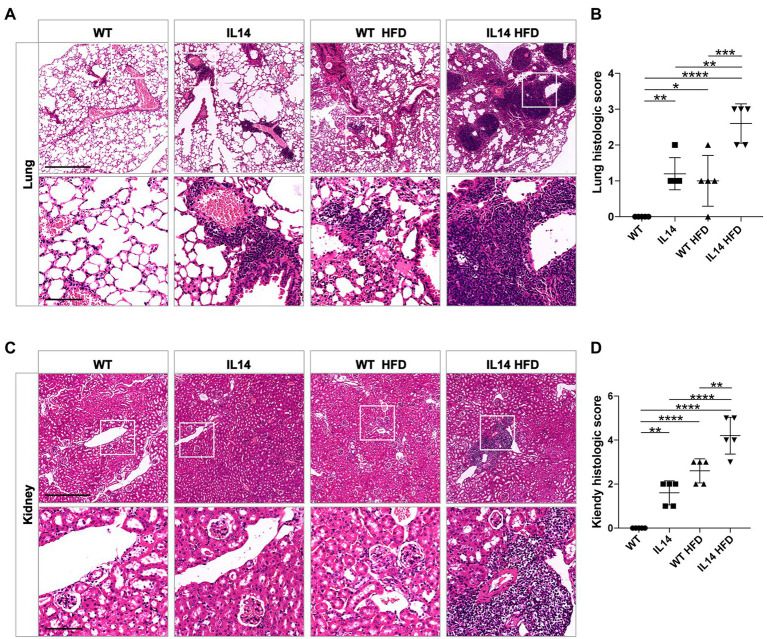
HFD exacerbated lung and kidney injury in the IL-14α TG mice. Lung and kidney tissues from the WT, IL14, WT HFD, and IL14 HFD groups were isolated for analysis. Lung **(A)** and kidney **(C)** sections were stained with H&E, and representative images are shown. **(B,D)** A histologic score of the lungs and the kidneys was quantified as described in the section “Materials and Methods,” showing worse tissue damage and an increase in infiltration in the IL14 HFD group. ^*^p ≤ 0.05; ^**^p ≤ 0.01; ^***^p ≤ 0.005; and ^****^p ≤ 0.001; *n* = 5 per group. Scale bars: 500 μm for H&E staining, 100 μm for enlarged images.

Our previous study showed that IL-14α TG mice developed interstitial lung and mild renal diseases after 14 months of age (Shen et al., 2013). Thus, pulmonary and renal pathology were investigated to explore the effect of HFD on pSS extra-glandular features. Histological evaluation of their kidneys and lungs revealed minimal lymphocytic inflammation and pathological change in the IL14 and WT HFD groups at 12 months of age. However, the lung and kidney showed severe lymphocytic infiltrates and higher histological scores in the IL14 HFD group at 12 months of age (Figures 4A–D). Those data indicated that HFD could promote systemic manifestations in the IL-14α TG mice.

### Taxonomic composition and diversity of gut microbiota analysis

The findings suggested that HFD exacerbated the evolution of pSS in the IL-14α TG mice. To determine whether gut microbiota played a role in this course, 16S rRNA sequencing was processed. A total of 971,604 quality-filtered sequences were obtained from the 20 analyzed samples after trimming and filtering, with an average of 48580.2 ± 4402.74 reads for each sample (Supplementary Table S1), and 560 OTUs were then clustered based on 97% sequence identity. In the taxonomic assignment process, these OUTs were binned in 11 phyla, 37 families, 61 genera, and 200 species.

**Figure 5:**
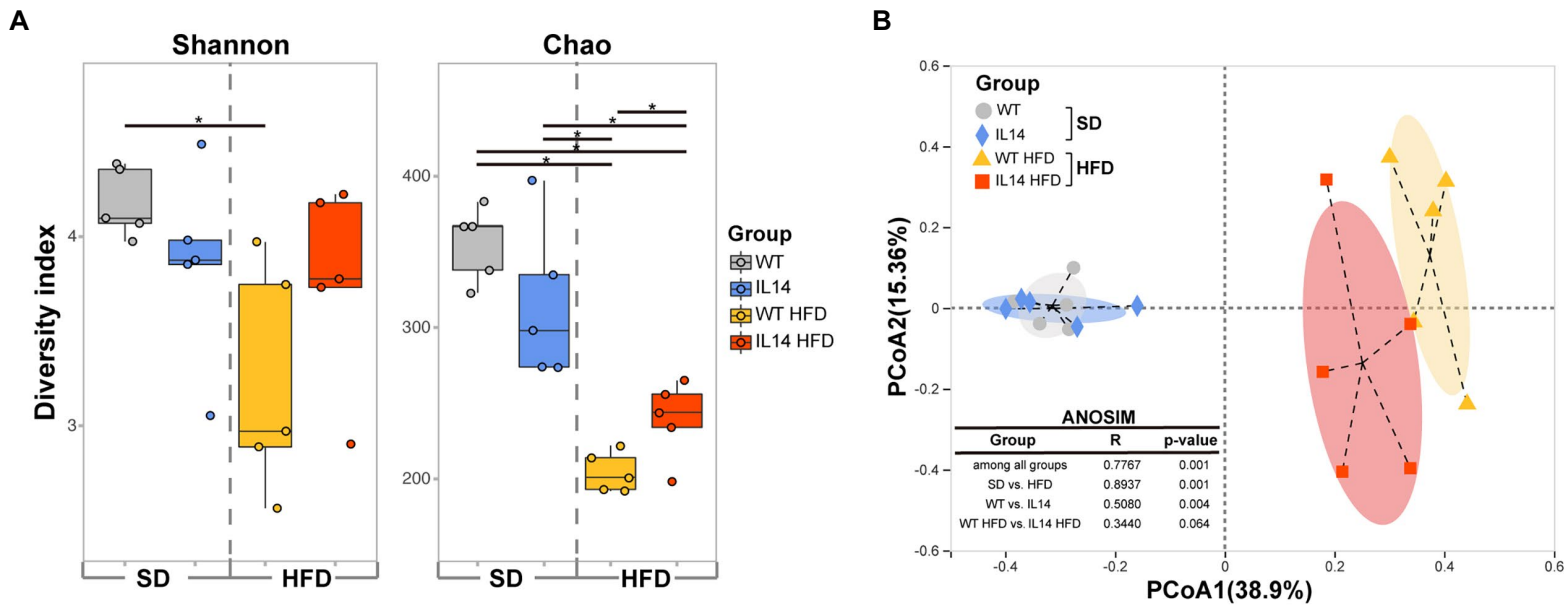
HFD altered diversity of gut microbiota in the IL-14α TG mice. **(A)** Alpha diversity comparison based on the Shannon diversity index and the Chao richness index with dot plots and box plots. **(B)** Principal coordinate analysis plot generated using operational taxonomic unit (OTU) metrics based on the Bray–Curtis dissimilarities. Each point represents a sample. ^*^p ≤ 0.05, *n* = 5 per group. SD, Standard diet; HFD, High-fat diet.

The richness and diversity of gut microbiota were assessed using alpha diversity. To compare the alpha diversity, the Shannon and Chao indices were calculated after rarefying the OTU table to 30,246 sequences, which is the size of the smallest sample to obtain equal sequencing depth. These analyses revealed a significant decrease in Shannon and Chao indices in the WT HFD group compared to the WT group (*p* < 0.05). Compared with the IL14 group, the Chao index of IL14 HFD was also significantly decreased (*p* < 0.05), but there was no significant change in the Shannon index (*p* > 0.05). Notably, after feeding HFD, the Chao index was significantly increased (*p* < 0.05) in the IL14 HFD group compared with the WT HFD group, while no significant difference was shown both in Shannon and Chao indices between the WT group and the IL14 group feeding SD (*p* > 0.05; Figure 5A).

A principal coordinate analysis (PCoA) was then conducted to visualize the obvious divergences in taxon composition among different groups (*R* = 0.7767, *p* = 0.001, ANOSIM; Figure 5B). The SD group was far away from the HFD group (*R* = 0.8937, *p* = 0.001), which indicated that the gut microbial communities of the HFD group had changed.

### HFD aggravated the dysbiosis of gut microbiota in IL-14α TG mice

**Figure 6:**
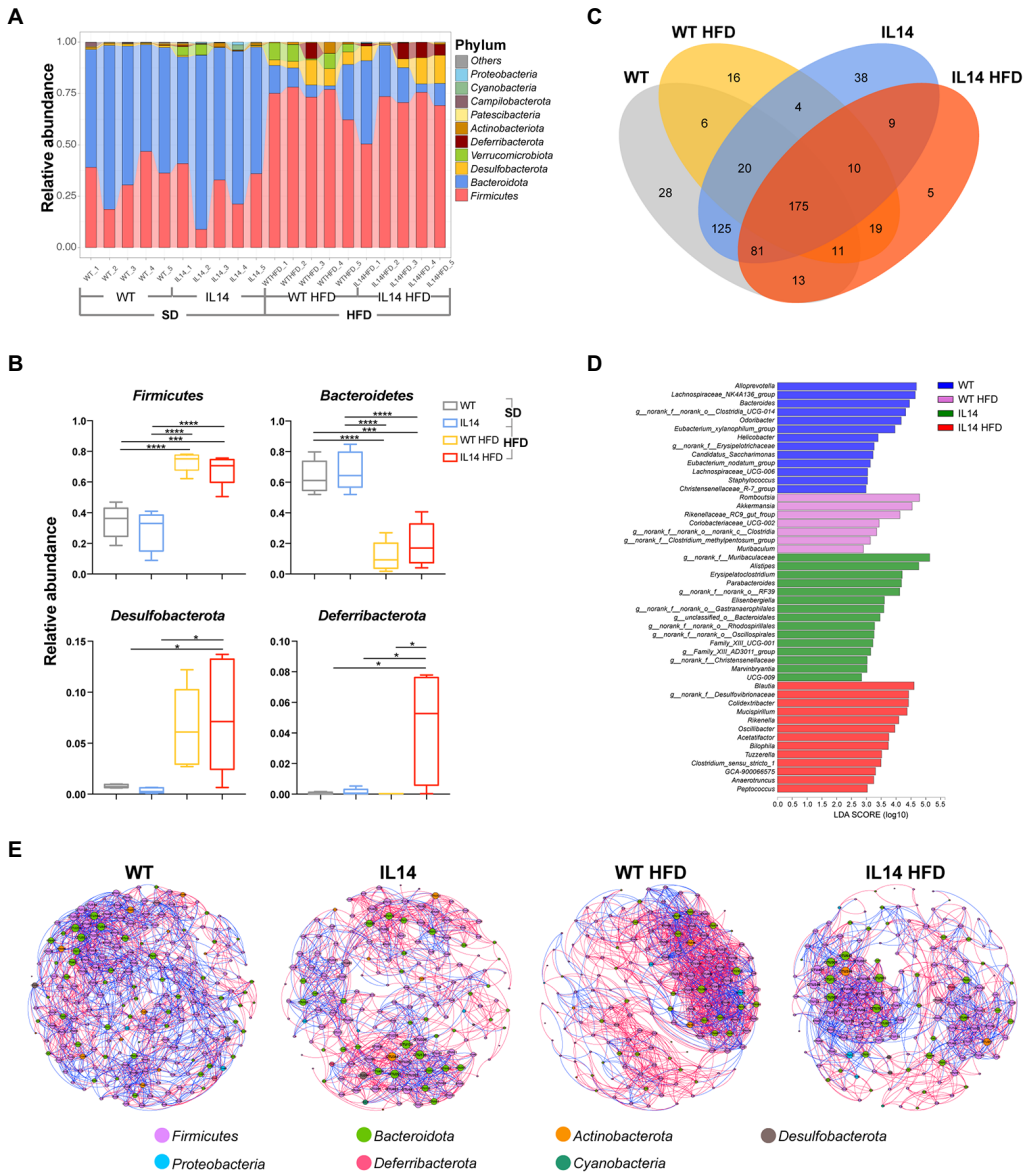
HFD affected the composition of the gut microbiota in the IL-14α TG mice. **(A)** Fecal microbial composition at the phylum level. **(B)** The relative abundance of the phyla *Firmicutes*, *Bacteroidetes*, *Desulfobacterota*, and *Deferribacterota* in the WT, IL14, WT HFD, and IL14 HFD groups. **(C)** A Venn diagram showing shared or unique OTUs among different groups. **(D)** Linear discriminant analysis (LDA) effect size (LefSe) analysis of bacterial communities with LDA scores greater than 2. Differences are represented by the color of the most abundant genus (blue is WT; pink is HFD; green is IL14; and red is IL14 HFD). **(E)** The networks of co-occurring bacterial OTUs in feces of four groups, based on the correlation analysis. The co-occurring networks are colored by phylum. A red edge indicates a positive interaction between two individual nodes, while a blue edge indicates a negative interaction. ^*^p ≤ 0.05; ^***^p ≤ 0.005; and ^****^p ≤ 0.001; *n* = 5 per group.

Community bar-plot analysis displays relative levels of the gut microbial community in all the 20 fecal samples at the phylum level (Figure 6A). A core microbiome consisting predominantly of *Firmicutes*, *Bacteroidetes*, *Desulfobacterota*, *Verrucomicrobiota*, *Deferribacterota*, and *Actinobacteriota* phyla was found, accounting for up to 90% of sequences on average. The remaining bacterial population (*Patescibacteria*, *Campilobacterota*, *Cyanobacteria*, and *Proteobacteria*) had a relative abundance lower than 1% in all groups.

To investigate the specific changes in gut microbiota, we compared the relative abundance of the predominant taxa identified from sequencing the four groups. The HFD group showed a significant decrease in the abundance of *Bacteroidetes* and a significant increase in the abundance of *Firmicutes* and *Desulfobacterota* compared with the SD group. We found that the IL14 HFD group showed a significantly enriched abundance of *Deferribacterota* compared with other groups (Figure 6B). It was also noted that 125 OTUs in fecal samples were shared between the WT group and the IL14 group, while only 19 OTUs were shared after high-fat feeding. In addition, only 9 OTUs were shared between the IL14 and IL14 HFD groups (Figure 6C). The results of the LEfSe analysis indicated that, compared to other groups, the relative abundance of *Blautia*, *norank_f_Desulfovibrionaceae*, *Colidextribacter*, *Mucispirillum*, *Rikenella*, *Oscillibacter*, *Acetatifactor*, and *Bilophila* were significantly enriched in IL14 HFD group, which have been reported as potentially pathogenic microorganisms (Figure 6D). We further compared the enriched genera in IL14 HFD with WT and IL14 groups, and the results observed that some of these genera, including *Colidextribacter*, *Mucispirillum*, and *Bilophila*, have an increased distribution with the severity of the disease. At the same time, the relative abundance of *norank_f__Muribaculaceae*, *Alloprevotella*, *Bacteroides*, *Alistipes*, and *Prevotellaceae_UCG-001* were significantly decreased in IL14 HFD (Supplementary Figure S1). Meanwhile, we compared the changes in gut microbiota in WT and IL14 mice under the high-fat diets; the results are illustrated in Supplementary Figure S2. At the phylum level, we found that the IL14 HFD group showed a significant decrease in the abundance of *Verrucomicrobiota* and an increased abundance of *Deferribacterota* and *Patescibacteria*. The results of the LEfSe analysis indicated that, after high-fat feeding, there was a significant increase in the number of significantly different genera between the WT HFD and IL14 HFD groups. Meanwhile, under high-fat diets, the results observed that some of these genera, including *Mucispirillum* and *Bilophila*, still increased in distribution with increasing disease severity in the IL14 HFD group.

We then explored the bacterial co-occurrence patterns among four groups using network analysis based on strong and significant correlations (Jiang et al., 2017b; Figure 6E). Overall, the ecological networks were markedly different among the different groups. Notably, there were more positive than negative correlations in all networks, regardless of aggregate fractions (Supplementary Table S2). The values of graph density, average clustering coefficient (avgCC), average degree (avgK), average weighted degree, and modularity in these empirical networks were significantly different among these groups, suggesting that the bacterial community composition was significantly different among each group. The co-occurrence patterns were further compared across four aggregate fractions, indicating that the bacteria community was more closely correlated (e.g., more abundant nodes) to each other in the WT group than in the IL14, WT HFD, and IL14 HFD groups. Furthermore, average path length and modularity were greater in the IL14 HFD group than in the other group networks except for the WT group. Further structural analysis showed that the deterministic pattern of intra-phylum co-occurrence was prevalent in the bacterial networks. The bacterial OTUs in the dominant phylum, *Firmicutes*, *Bacteroidota*, *Actinobacteriota*, and *Proteobacteria* tended to co-occur more than other phyla.

### HFD increased the correlation between gut microbiota and clinical parameters in the IL-14α TG mice

**Figure 7:**
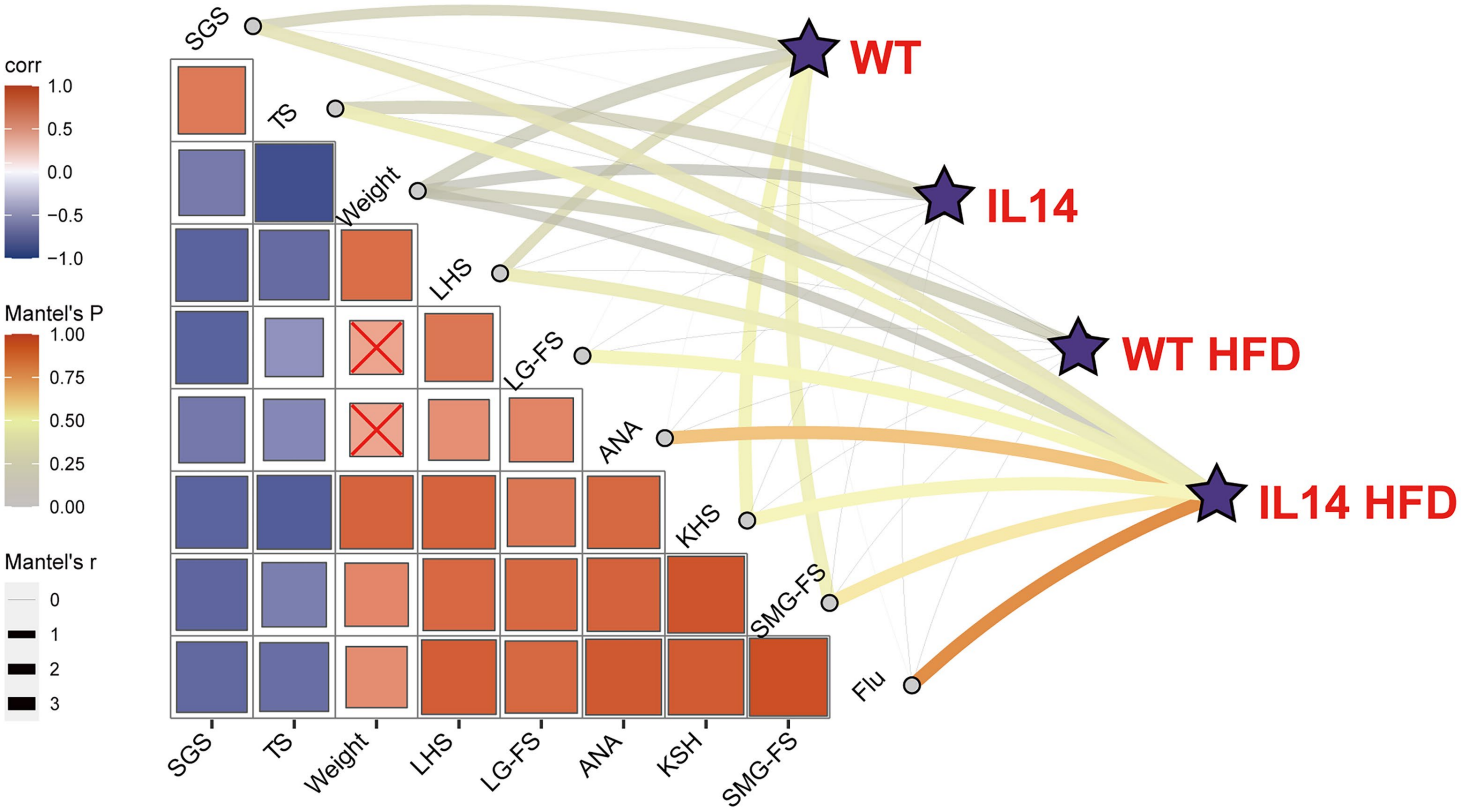
Correlation between gut microbiota and clinical parameters. The correlation between gut microbial communities (Bray-Curtis distance) and clinical parameters in different groups was analyzed with the Mantel tests. The edge width corresponds to the *R*-value, and the edge color denotes the statistical significance. The color gradient indicates Pearson correlation coefficients among the clinical parameters × indicates no significant correlation at the 0.05 level. SGS, salivary gland secretions; TS, tear secretions; LHS, lung histologic score; LG-FS, lacrimal gland focus score; ANA, antinuclear antibodies; KHS, kidney histologic score; SMG-FS, submandibular gland focus score; and Flu, fluorescein staining score.

We further examined whether there was a correlation between gut microbiota and clinical parameters in four groups using the Mantel tests (Figure 7). The gut microbial communities exhibited a significant correlation with SGS, weight, SG-FS, and LHS in the WT group. The gut microbial communities in IL14 exhibited a significant correlation with TS and weight, while the WT HFD group only exhibited a significant correlation with weight but showed no correlation with others. Interestingly, the gut microbial community composition of the IL14 HFD group was significantly correlated with all clinical characterization, such as all ocular surface-related indicators (Flu, TS, LG-FS), ANA, SGS, and SMG-FS. This phenomenon further confirmed that the gut microbial communities of the IL-14α TG mice were specifically correlated with clinical parameters after HFD-feed.

## Discussion

Previous studies showed that HFD was found to exacerbate the development of autoimmune disease (Swanson et al., 1989); however, the pathogenesis was still less understood. Here in, we showed that the dysbiosis of gut microbiota induced by HFD was one of the primary causes of the disease exacerbation in IL-14α TG mice. Moreover, HFD resulted in increased potentially pathogenic microbial exposure, which exacerbated the clinical manifestations in IL-14α TG mice, including the declined secretions of the salivary gland and increased focus score of submandibular and lacrimal glands.

Microbiota diversity is essential to maintaining ecosystem balance. Previous study demonstrated that the severity of SS ocular and systemic diseases was inversely correlated with microbial diversity (de Paiva et al., 2016). In a clinical investigation, most studies showed the decreased alpha-diversity in pSS (Mandl et al., 2017; van der Meulen et al., 2019; Wu et al., 2019). One study found no significant difference (Moon et al., 2020), while another study found no difference in Shannon’s diversity index, but Faith’s phylogenetic diversity showed an increased diversity in pSS (Mendez et al., 2020). Regarding alpha diversity, lower richness in gut microbiota composition was present in the IL14, WT HFD, and IL14 HFD groups compared to the WT group. Although the abundance of gut microbiota was higher in the WT group than in the IL14 group, no statistical significance among the groups may be due to IL-14α TG mice developing autoantibodies, sialadenitis, as in SS at the age of 9–17 months (Shen et al., 2006), and the IL-14α TG mice used in the experiment had only initial manifestations, such as there were no differences of SGS, ANA, TS, and LG-Fs between the WT and IL14 groups. The Shannon index was significantly lower only in the WT HFD group than in the WT group, suggesting that the diversity of gut microbiota in IL-14α TG mice was not affected by HFD. In addition, the Bray-Curtis PCoA plot for beta diversity showed that the HFD group was grouped into a tight cluster compared to the ND group. These results suggested that the abundance and beta diversity of gut microbiota was significantly affected in IL-14α TG mice under the state of high-fat feeding.

Members of the *Bacteroidetes* and *Firmicutes* phyla dominate the gut microbiota, and the microbiota of an individual is more similar to self over time than to others (Human Microbiome Project Consortium, 2012). It is well-known that HFD can cause disturbances in gut microbiota, but the obese mouse/human fecal community has been found to have a significantly higher relative abundance of *Firmicutes* (Anhe et al., 2015; Chang et al., 2015; Singh et al., 2017) and *Desulfobacterota* (Wei et al., 2021) and a lower relative abundance of *Bacteroidete* (Anhe et al., 2015; Chang et al., 2015; Singh et al., 2017), which was consistent with our results. Moreover, at the phylum level, we observed that the relative abundance of *Deferribacterota* was significantly increased only in the IL14 HFD group. The results of LefSe further indicate that *Mucispirillum* was enriched in the IL14 HFD group, which belongs to the phylum *Deferribacterota* compared with other groups*. Mucispirillum* is, as of today, the only genus of the *Deferribacteraceae* known to inhabit the vertebrate gastrointestinal tract (Harrell et al., 2012; Rosshart et al., 2017; Michel et al., 2018; Umu et al., 2020). Due to its low relative abundance in human fecal samples, it has not been noted in the majority of human studies (Herp et al., 2021). A large number of studies linked *Mucispirillum* to inflammatory bowel disease (IBD; Kabbert et al., 2020; Herp et al., 2021) and a variety of other diseases (Belzer et al., 2014). Moreover, the increase of *Mucispirillum* has been linked to HFD (Ussar et al., 2015), stress (Jasarevic et al., 2017), and diseases like rheumatoid arthritis (Xiao et al., 2018) or Parkinson’s disease (Lin et al., 2019). It was also reported that *Mucispirillum* was positively related to the autoimmune diseases activity and that the abundance could be an indicator of collagen induced-arthritis (Ben-Amram et al., 2017). Therefore, *Mucispirillum* may play an important role in the development of Sjögren’s syndrome. In addition, at the genus level, we further observed that HFD could significantly increase the relative abundance of potentially pathogen genera in IL-14α TG mice. Like *Mucispirillum*, there were three other genera associated with autoimmune disease activity. The positive correlations between *Oscillibacter*, *Bilophila*, *Rikenella*, and autoimmune disease activity were in accordance with a prior study. Not only that, the three genera showed significant increases from the predisease stage to the diseased stage (Luo et al., 2018). In the results, we similarly found that the relative abundance of *Mucispirillum* and *Bilophila* was significantly increased from a mild to a severe stage in IL-14α TG mice. *Blautia*, *norank_f_Desulfovibrionaceae*, and *Colidextribacter* have been reported as HFD-dependent taxa (Li et al., 2022; Ozato et al., 2022). According to previous studies, *Acetatifactor* might be associated with the development of non-alcoholic fatty liver disease induced by HFD (Zhang et al., 2021). Therefore, we postulate that HFD may aggravate the condition of Sjögren’s syndrome mice by increasing potentially pathogenic microorganisms in the intestine. Furthermore, these findings showed that *Bacteroides*, *Alistipes*, *Prevotellaceae_UCG-001*, and *Parabacteroides* weresignificantly lower in IL14-α TG mice after HFD-feed, which was consistent with previous clinical studies of SS (de Paiva et al., 2016; Cano-Ortiz et al., 2020).

Of note, the results observed that there was almost no presence of *Verrucomicrobiota* in the WT group under the standard diets until the old state, and the IL14 group showed an increase in the abundance of *Verrucomicrobiota* compared with the WT group. In a previous report, which is similar to our results, the abundance of *Akkermansia* of Phylum *Verrucomicrobia* was significantly increased in SS mice compared with the control (Lu et al., 2020). However, after feeding high-fat diets for a long time, the IL14 HFD group showed the absence of *Verrucomicrobiota* compared with the WT HFD group. In addition, at the genus level, we found that the abundance of *Akkermansia* increased in the WT HFD group as compared with the WT group. Our results are in contrast to previous literature (Zhu et al., 2018; Chen et al., 2020), which may be age-related. Clea Bárcena et al. found that fecal microbiota transplantation from wild-type mice enhanced healthspan and lifespan in progeroid mouse modelsand that transplantation with *Verrucomicrobia Akkermansia muciniphila* was sufficient to exert beneficial effects, suggesting the existence of a link between aging and the gut microbiota (Bárcena et al., 2019). Also, *Akkermansia* aggravated the intestinal inflammation induced by *Salmonella typhimurium* and destroyed the microbial balance of the intestinal mucosa of the host (Ganesh et al., 2013). Meanwhile, the abundance of *Akkermansia* was not different in the WT, IL14, and IL14 HFD groups, indicating that the abundance of *Akkermansia* does not change with disease severity; it may be due to the very low abundance situation in all three groups. Therefore, the change in the abundance of *Akkermansia* may be affected by age, genotype, and diet.

To demonstrate whether HFD aggravated the condition of the mice, we used IL-14α TG mice with mild pSS symptoms. pSS is a systemic autoimmune disease and is characterized by lymphocytic infiltration of the exocrine glands (Doare et al., 2021), in particular, the salivary and lacrimal glands, resulting in oral and ocular dryness complaints (Mariette and Criswell, 2018). In this study, as expected, an HFD can increase the lymphocytic infiltration of lacrimal and submandibular glands of IL-14α TG mice, along with a significant decrease in the secretions of the salivary gland. Similarly, Xin He et al. demonstrated an HFD-induced lipid accumulation in the lacrimal gland, which led to a series of pathologic changes in the lacrimal gland acinar that reduced lacrimal gland aqueous tear secretion (He et al., 2020). Yang Wu found that an HFD induces dry eye-like ocular surface damages in mice *via* the activation of oxidative stress and induction of apoptosis in the cells of the ocular surface (Wu et al., 2020). In addition, Swanson et al. revealed that lipid intake exacerbates the progression of autoimmune disease, which was indicated by increased infiltration of inflammatory cells into the exocrine tissue (Swanson et al., 1989). Based on our results, we found a significant positive association between the clinical parameters and the abundance of gut microbiota in the IL-14α TG mice after HFD feeding.

We also noticed that HFD alone could induce some symptoms associated with pSS. Previous studies showed that HFD induced lipid accumulation in the lacrimal gland after 1 month on the HFD. Lipid deposition in the lacrimal gland led to lower lacrimal gland secretions, fewer immune cell infiltration, and an increase in inflammation-related cytokines levels and apoptosis cells. HFD alone could induce a series of pathologic changes in the lacrimal gland (He et al., 2020). However, HFD failed to induce lipid deposition in the salivary gland (de Carvalho et al., 2017). On the other hand, the saliva secretions were also regulated by parasympathetic and sympathetic innervations (Proctor and Carpenter, 2014). Tyrosine hydroxylase, a useful marker of sympathetic nerves, significantly decreased after 2 and 3 months of HFD in submandibular glands (de Carvalho et al., 2017), which may explain the decrease in gland secretions. Additionally, HFD could perturb B-cell production, activity, and maturation by changing the composition of the gut microbiome (Shaikh et al., 2015; Petta et al., 2018). Previous studies showed that autoreactive and pro-inflammatory antibodies were increased in obese humans and HFD-fed mice (Rosenbloom, 2003; Marzullo et al., 2010). All of these likely findings help to account for how the HFD caused these changes in WT animals.

Findings from this study should be interpreted while bearing in mind that our study has the limitation of a relatively small number of experimental animals. In addition, due to the gut having a wide diversity of bacterial flora, we could completely construct the intestinal ecological environment similar to SS patients.

## Conclusion

Our data suggest that HFD exacerbated the pSS symptom in the IL14α TG mice. The worse pSS clinical parameters in the IL14α TG mice were associated with the high relative abundance of potentially pathogenic genera induced by HFD.

## Data availability statement

The datasets presented in this study can be found in online repositories. The names of the repository/repositories and accession number(s) can be found at: NCBI—PRJNA833247.

## Ethics statement

The animal study was reviewed and approved by Experimental Animal Ethics Committee of Xiamen University.

## Author contributions

GS supervised the whole project. LS and MZ conceived the project. YicL conducted the animal and H&E experiments. YaL conducted 16S rRNA sequencing and sequencing data analysis. YixL conducted an ANA test. MZ and YaL conducted the image processing and analysis. All authors contributed to the article and approved the submitted version.

## Funding

The work was supported by the Natural Science Foundation of China (grant no. 81971536, U1605223, and 82171779) to GS, and the Xiamen Health Commission (grant no. 3502Z20209004) to GS.

## Conflict of interest

The authors declare that the research was conducted in the absence of any commercial or financial relationships that could be construed as a potential conflict of interest.

## Publisher’s note

All claims expressed in this article are solely those of the authors and do not necessarily represent those of their affiliated organizations, or those of the publisher, the editors and the reviewers. Any product that may be evaluated in this article, or claim that may be made by its manufacturer, is not guaranteed or endorsed by the publisher.

## Supplementary material

The Supplementary Material for this article can be found online at: https://www.frontiersin.org/articles/10.3389/fmicb.2022.916089/full#supplementary-material

## Footnotes

^1^ http://huttenhower.sph.harvard.edu/LEfSe
