# Supplementary material for: High-fat diet-induced intestinal dysbiosis is associated with the exacerbation of Sjogren’s syndrome
Source: Front Microbiol. 2022 Jul 22;13:916089. doi: 10.3389/fmicb.2022.916089 (PMC9354669; doi:10.3389/fmicb.2022.916089)
Supplement: Supplementary file 1 [file Data_Sheet_1.docx]

**Table S1. Summary statistics of 16S rRNA gene sequencing**

| **Segments** | **N** | **Clean reads** | **coverage range** | **Average of sequences per sample** |
| --- | --- | --- | --- | --- |
| **WT** | 5 | 259488 | 98.0824 | 51897.6 |
| **IL4** | 5 | 238416 | 98.2832 | 47683.2 |
| **WT HFD** | 5 | 234421 | 98.2702 | 46884.2 |
| **IL14 HFD** | 5 | 239279 | 98.097 | 47855.8 |
| **Total** | 20 | 971604 | 98.1832 | 48580.2 |

N = the number of non-missing values;

**Table S2. Topological properties of co-occurring bacterial networks obtained among four groups (WT, IL14, WT HFD, IL14 HFD)**

| **Network metrics** | **Total bacterial community** | | | |
| --- | --- | --- | --- | --- |
| **Empirical networks** | **WT** | **IL14** | **WT HFD** | **IL14 HFD** |
| **Number of nodes** | 223 | 157 | 149 | 145 |
| **Number of edges** | 1978 | 1223 | 1616 | 1252 |
| **Number of positive correlations** | 58.54% | 72.85% | 79.21% | 66.61% |
| **Number of negative correlations** | 41.46% | 27.15% | 20.79% | 33.39% |
| **Average path length (APL)** | 3.53 | 3.59 | 3.48 | 3.64 |
| **Graph Density** | 0.08 | 0.1 | 0.147 | 0.12 |
| **Network diameter** | 7 | 10 | 10 | 9 |
| **Average clustering coefficient (avgCC)** | 0.621 | 0.668 | 0.68 | 0.673 |
| **Average degree (avgK)** | 17.74 | 15.58 | 21.691 | 17.269 |
| **Modularity (M)** | 6.006 | 1.28 | 0.814 | 1.908 |
| **Average Weighted Degree** | 3.294 | 7.803 | 14.362 | 6.539 |

**
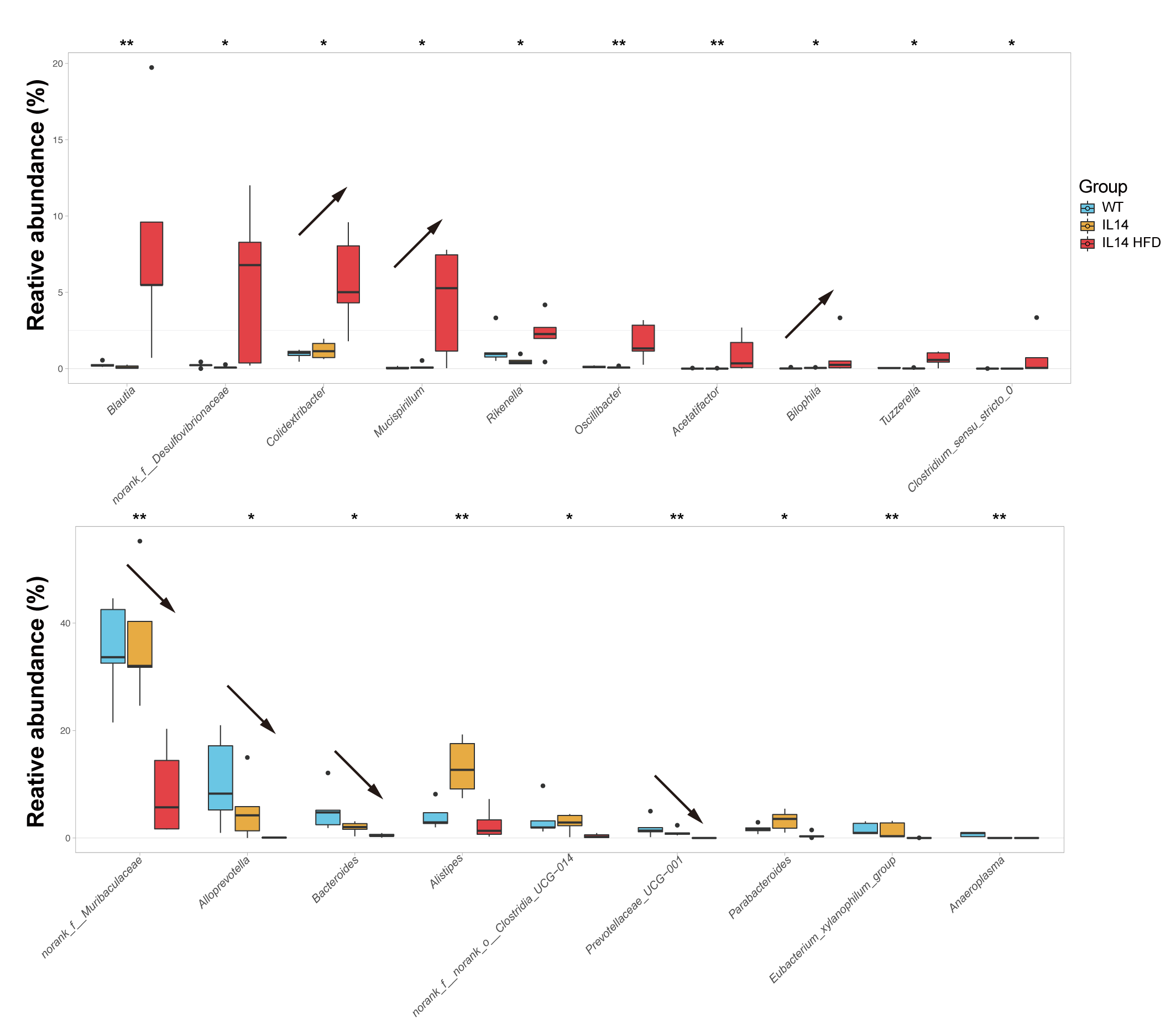
**

**Figure S1.** **Comparison of significant shifts in the abundance of genera between WT, IL14 and IL14 HFD groups.** Significant genera with trend change were indicated by arrows that increase (bottom) or decrease (top). *P ≤ 0.05; **P ≤ 0.01; ***P ≤ 0.005; and ****P ≤ 0.001; n=5 per group.


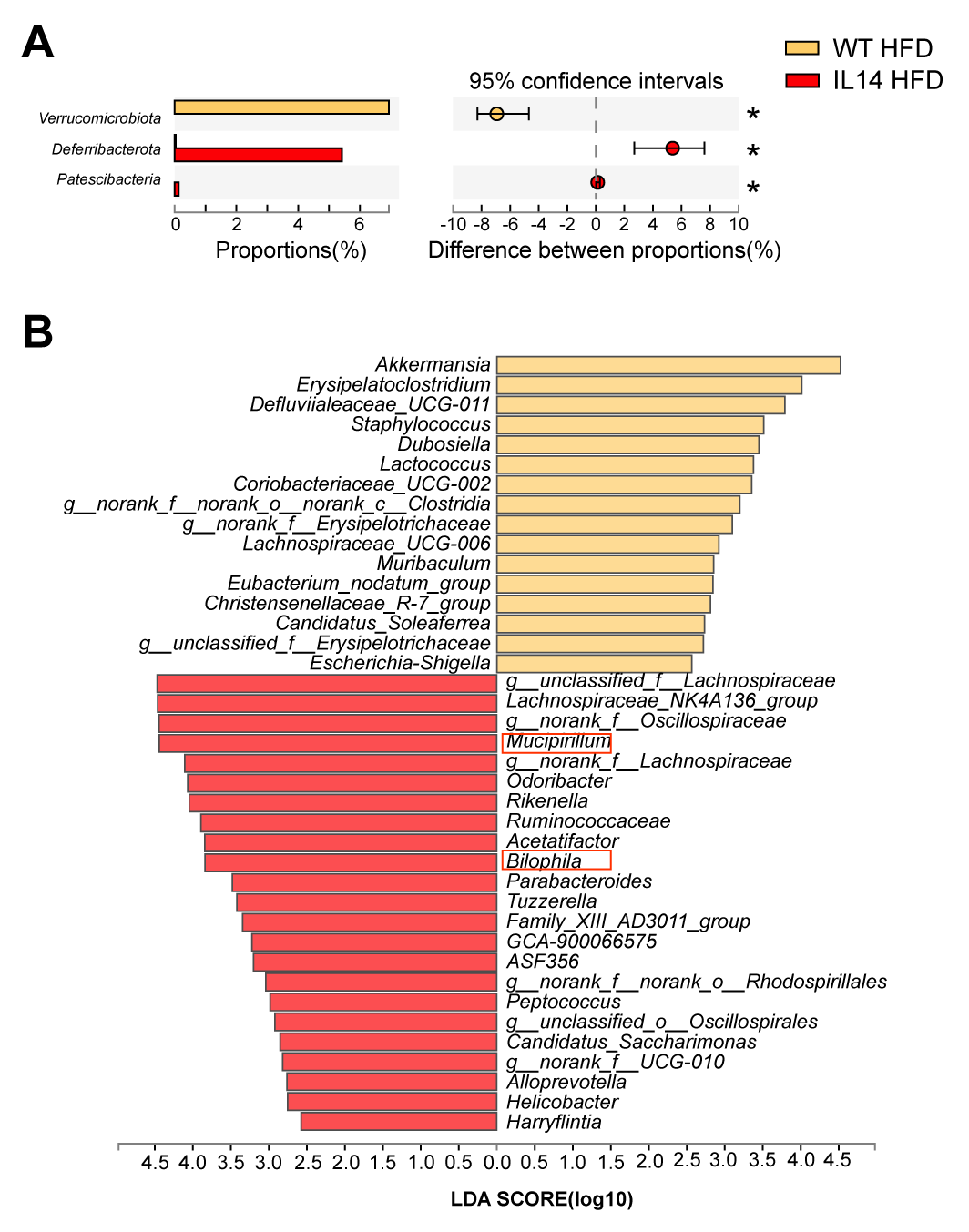


**Figure S2. Comparison of significant shifts in the abundance of phylum and genus between WT HFD and IL14 HFD groups.** (**A**) The significant shifts between WT HFD and IL14 HFD groups at phylum level (*P ≤ 0.05; **P ≤ 0.01; ***P ≤ 0.005; and ****P ≤ 0.001; Mann-Whitney U test, n=5 per group). (**B)** LEfSe analysis of bacterial communities with LDA scores greater than 2. Differences are represented by the color of the most abundant genus (yellow is WT HFD; red is IL14 HFD).
